# Pulmonary artery pseudoaneurysms: a single-center experience of endovascular occlusion

**DOI:** 10.1186/s42155-023-00411-9

**Published:** 2023-12-02

**Authors:** Adam Fish, Anne Sailer, Jeffrey Pollak, Todd Schlachter

**Affiliations:** Department of Interventional Radiology, Yale School of Medicine, New Haven, USA

## Abstract

The technique and outcomes of pulmonary artery pseudoaneurysm (PAP) embolization was retrospectively evaluated in 13 patients undergoing 14 PAP embolizations between January 2014 and September 2023. The etiology of the PAP was iatrogenic (4/13), tumor (3/13), chronic lung (2/13), idiopathic (2/13) and mycotic (2/13). Clinical presentation was massive hemorrhage (6/13), incidental (4/13), and non-massive hemoptysis (3/13). The average PAP size was 13.5 mm. Coil embolization of the PAP sac was performed in all but two extenuating cases (11/13). Follow-up of 12 patients over an average 5.3-months showed persistent occlusion in all cases. There were no major adverse events attributed to the embolization. Five out of ten patients with procedures performed at least one year before this study were noted to be deceased after an average seven-month time. PAPs of various etiologies may be safely and effectively treated by occluding the aneurysm inflow, outflow, and sac.

## Introduction

Pulmonary artery pseudoaneurysms (PAPs) are life-threatening lesions arising from traumatic, iatrogenic, infectious, inflammatory, or neoplastic injury to the pulmonary arteries. It is the etiology of hemoptysis in < 10% of cases [1], resulting in an 87% rupture rate if left untreated [2]. Multiple small studies have shown catheter directed embolization to be an effective treatment option for PAPs [1, 3, 4], however these studies largely focused on the treatment of mycotic aneurysms. Shin et al [1] described four types of pulmonary pseudoaneurysms, all in the setting of infection, based on the presence of systemic artery collateralization, with treatment addressing PAPs that are perfused retrogradely from the systemic arterial circulation. Chen et al [3] described the imaging characteristics of 35 PAPs of multiple etiologies in twelve patients, concluding PAPs show a predilection for peripheral pulmonary arteries, and multiplicity of PAPs are commonly seen in endocarditis and pulmonary metastatic disease. Though these limited studies are helpful with the characterization of PAPs and treatment of those with bronchial artery origin, there are no large case series evaluating treatment of PAPs without systemic supply. Although pseudoaneurysms are treated throughout the body by a standard front-door, back-door approach, there is a concern that a similar approach which includes embolization of the PAP sac may rupture [4]. The aim of this study was to assess the efficacy and safety of endovascular treatment of pulmonary artery pseudoaneurysms arising from multiple etiologies by embolizing the pseudoaneurysm sac with permanent embolic agents.

## Materials and methods

With institutional review board approval, a single-institution imaging database search was performed for pulmonary artery pseudoaneurysms between January 2014 and September 2023. Thirteen patients that underwent embolization for pulmonary artery pseudoaneurysm were identified. Patient characteristics, clinical presentation and CT findings were recorded. Lesion size and embolization agents and techniques were documented, including whether occlusion of the pulmonary arterial inflow, aneurysmal sac, and/or outflow was achieved. Technical success was defined as complete cessation of aneurysmal sac opacification. Post-procedural outcomes including recurrent hemoptysis, adverse events and long-term survival were documented. Clinical success was defined as cessation of hemoptysis resulting from pseudoaneurysm rupture or reperfusion.

## Results

Thirteen patients with a total of 14 pulmonary artery pseudoaneurysms were treated endovascularly. This included eight males with an average age of 60.8 years (age range, 39–84). Etiologies included iatrogenic injury in four patients (4/13), neoplasm (3/13), chronic inflammatory lung disease (2/13), mycotic (2/13), and idiopathic (2/13). Massive hemorrhage, defined as approximately > 250 cc of blood in 24 hours [5], was the presenting symptom in six patients (6/13). Three patients presented with non-massive hemoptysis (3/13) and asymptomatic incidental lesions were found on CTA in four patients. Of the four asymptomatic patients, two had histories consistent with PAP (right heart catheterization and chronic lung disease) with prior chest CTs demonstrating sub-acute changes. The other two asymptomatic patients did not have histories consistent with either pseudoaneurysm or true aneurysm. Eleven of the thirteen patients had pre-procedural imaging including CTA demonstrating pulmonary artery pseudoaneurysm in 5/11 patients, routine CT demonstrating a vascular lesion in 4/11 patients and a lung tumor without clear PAP in 2/11 patients. In 2/13 patient’s pre-procedural imaging was circumvented due to emergency of the procedure, secondary to massive hemoptysis. Lesion location involved the lower lobes in 9/14 lesions (5 RLL, 4LLL), four upper lobe lesions (2 RUL, 2 LUL) and one lesion originating from the left main pulmonary artery, secondary to tumor involvement. The average pseudoaneurysm sac size was 13.5 mm (8-30 mm). Table 1 summarizes the patient and lesion characteristics.

**Table 1:** Summary of patient histories and lesion characteristics

ID	Age	Sex	Etiology of PSA	Co-morbidities	Presentation	CT Diagnosis	Location	Size (mm)
1	70	M	Iatroenic	Heart failure, Afib	Incidental	Vascular lesion	LLL posterior segment	16
2	59	F	Chronic lung disease	ILD, Pul HTN	Incidental	PSA—CTA	LUL	22
3	86	M	Iatroenic	Afib, Valvular disease	Massive hemorrhage	None	RLL	8
4	84	F	Iatroenic	COPD, AS s/p TAVR	Hemoptysis requiring airway protection	PSA—CTA	RML	30
5	60	F	Iatroenic	Heart transplant	Massive hemorrhage	None	RML	12
6	61	M	Tumor	COPD, Lung cancer	Massive hemorrhage	Lung mass	Left main	N/A
7	56	M	Tumor and/or radiation	COPD, Lung cancer	Massive hemorrhage	PSA—CTA	LUL	8
8	47	M	Tumor	Lung cancer	Massive hemorrhage	Cavitary mass	RLL	12
9	45	M	Idiopathic	None	Incidental	Vascular lesion—CT	LLL	11
10	39	F	Idiopathic	None	Incidental	Vascular lesion—CT	LLL	10
11	69	M	Chronic lung disease	COPD	Hemoptysis, small volume	Vascular lesion—CT	LLL	10
12	53	M	Mycotic	Endocarditis, IVDU	Hemoptysis requiring airway protection	PSA—CTA	RLL × 2	14, 10
13	62	F	Mycotic	Endocarditis, Fungemia	Massive hemorrhage	PSA—CTA	RLL	9

**Fig. 1:**
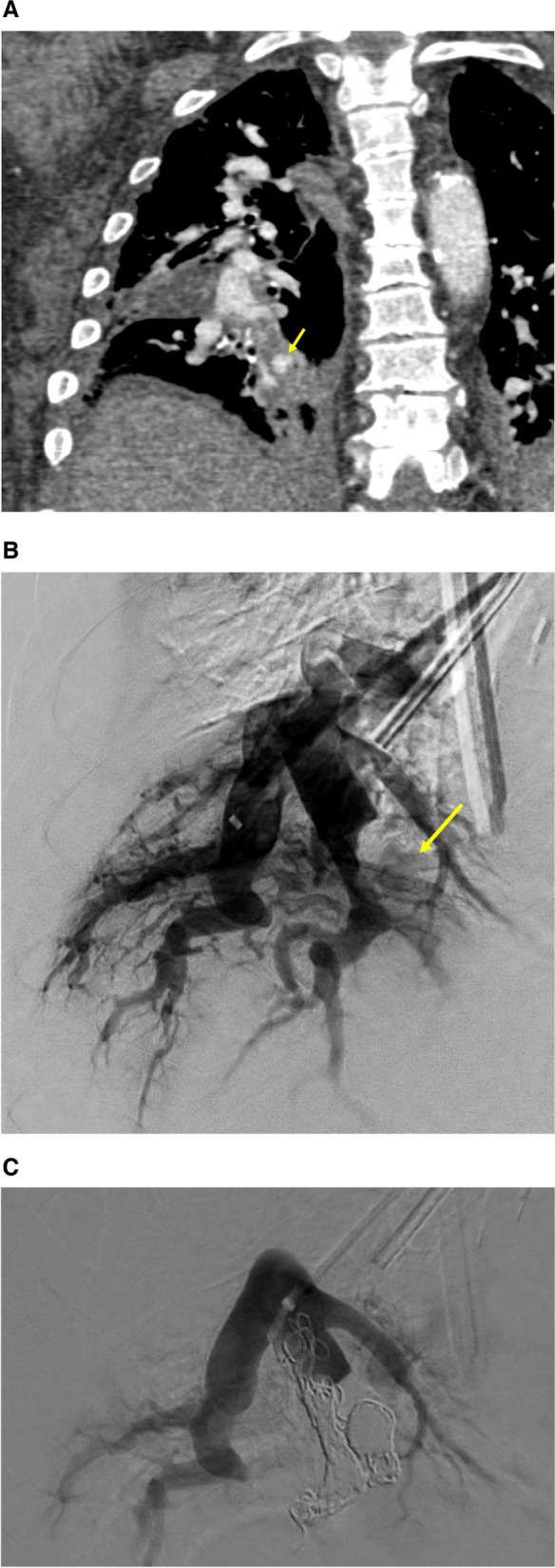
**a** Patient 13: A 62 year old woman with candidemia and endocarditis who presented with a partially thrombosed 1 cm pseudoaneurysm seen in the RLL. **b** Same patient. Selection of the right lower lobe with a 5 Fr 90 cm introducer sheath and 5 Fr 100 cm Barenstein catheter. Subtraction angiogram, AP, demonstrates faint filling of a PAP (yellow arrow) originating from an irregular RLL posterior segment, with questionable contribution from the medial segment. **c** Same patient. Now status post coil embolization of RLL posterior segment inflow, outflow, and sac, with multiple 8 and 10 mm Ruby microcoils advanced through a 2.4 Fr Progreat microcatheter

**Fig. 2:**
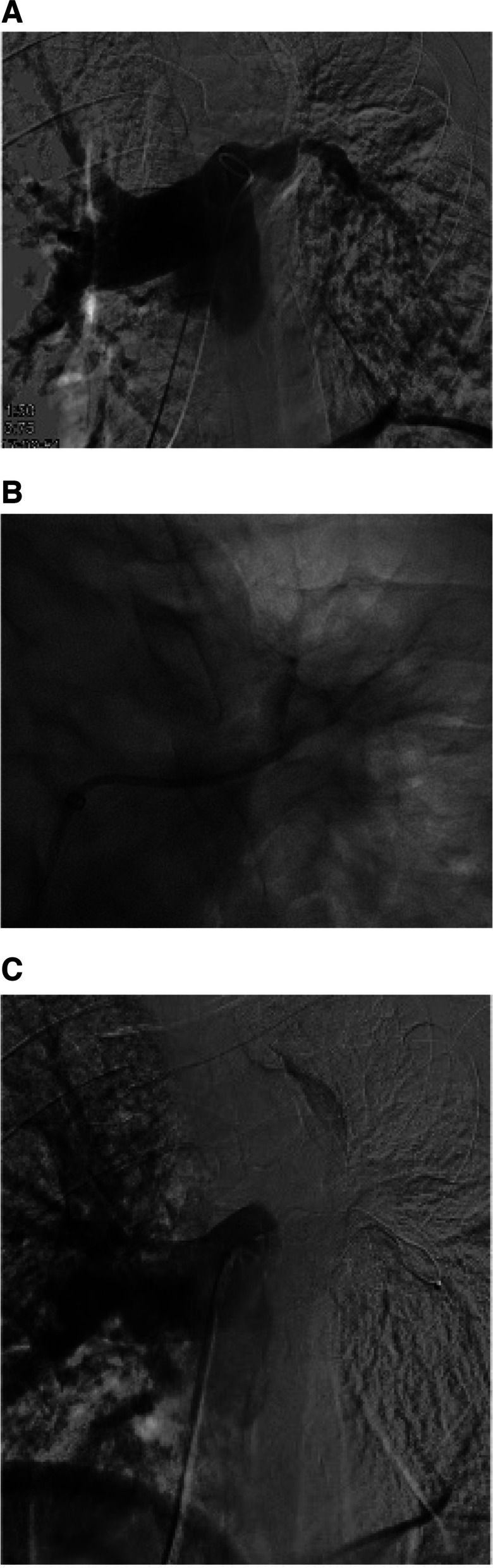
**a** Patient 6: A 61 y/o male with a large lung mass eroding into the left main pulmonary artery causing massive hemorrhage. Left main pulmonary angiogram demonstrating loss of integrity of the left main pulmonary artery. **b** Same patient. Selective angiogram of the left upper lobe after 350–500 mcm PVA embolization. **c** Same patient, now following occlusion of the left main pulmonary artery with a 22 mm Amplatzer Vascular Plug version 2 placed across the left main pulmonary artery aneurysm. Main pulmonary artery angiogram demonstrating complete occlusion of the left main pulmonary artery

Diagnostic pulmonary angiogram identified all lesions. The most common embolization technique involved embolization of the pulmonary artery inflow (within 1 cm of the sac), aneurysmal sac, and outflow, performed in 7/13 patients (7/14 lesions). Figure 1a-c demonstrates the inflow, sac, and outflow embolization technique. Embolization of the inflow and sac was performed in 3/13 patients (4/14 lesions), sac only in 1 case, and pulmonary arterial inflow only in 1 case. In one case (1/13), a large PAP of the left main pulmonary artery caused by a lung neoplasm was embolized with a large Amplatzer plug and PVA (Fig. 2a-c). Permanent embolic devices were used in all patients and cases (13/13 and 14/14 respectively), with the addition of PVA in 3/13 patients. Technical success was achieved in all patients and lesions (13/13, 14/14). Twelve of thirteen patients had follow-up, with one death occurring following the embolization procedure due to ventricular tachycardia. The 30-day mortality and inpatient mortality was 15.4% (2/13). Follow-up time periods ranged from 13 days to 20 months with an average follow-up time-period of 5.3 months. There was one case of minor recurrent hemoptysis secondary to reperfusion from a small collateral pulmonary arterial vessel. This was subsequently treated with PVA resulting in complete occlusion and no recurrent hemoptysis. Clinical success was therefore achieved in all patients (100%) after an average of 1.1 embolizations per lesion. Two additional patients presented with recurrent minor hemoptysis secondary to a lung neoplasm which was successfully treated with bronchial artery embolization. However, the PAP was shown to be occluded in these two cases. There were no major adverse events related to the embolization procedure; the patient that died was due to the underlying poor clinical condition rather than the procedure itself as they were both status post recent cardiac catheterization and presenting with massive hemoptysis. In one case, migration of the coils into the bronchi due to a neoplastic bronchopulmonary fistula occurred several months post-procedurally, however repeat pulmonary angiogram demonstrated persistent occlusion of the PAP. Minor self-resolving pleuritis occurred in 2/13 patients. Table 2 summarizes procedure technique and outcomes. Of note, 5/10 patients with procedures performed at least one year before the time of this study were noted to be deceased over an average 7-months post-procedure (0–14-month range).

**Table 2:** Summary of embolization technique, embolic agent and outcome

ID	Embolization technique	Embolic agent	Number of embolizations	Reperfusion	Post embo Hemoptysis	Adverse events	Last follow-up date
1	Outflow, Sac, Inflow within 1 cm of sac	Coils	1	No	No	Pleural effusion	5/16/22 (6 months)
2	Outflow, Sac, Inflow within 1 cm of sac	PVA outflow, coil sac and inflow	1	No	No	None	1/30/19 (20 months)
3	Outflow, Sac, Inflow within 1 cm of sac	Coils, Plug across the PSA, Coils prox	1	No	No	None/Death from Vtach	None
4	Sac only	Framing and packing coils	1	No	No	None	2/2/2022 (4 months)
5	Inflow only	Coils	1	No	No	None	7/14/2023 (2 mo)
6	Distal left main pulmonary artery	PVA and Plug of distal main LPA	1	No	No	None	5/30/18 (4 mo)
7	Sac and inflow	Coils	1	No	Yes^a^	Coil migration into airways	6/12/2022 (10 mo)
8	Outflow, Sac, Inflow within 1 cm of sac	Coils	1	No	Yes^a^	None	5/20/2014 (3 m/o)
9	Outflow, Sac, Inflow within 1 cm of sac	Coils, Coils, Plug inflow	1	No	No	Pleurtic chest pain	11/21/18 (9 m/o)
10	Sac and inflow	Coils and Plug inflow	1	No	No	None	9/14/18 (7 m/o)
11	Outflow, Sac, Inflow within 1 cm of sac	Coils; PVA of inflow on repeat	2	Yes—Minimal	Yes—None after 2nd	None	9/4/2023 (2 m/o)
12	Sac and inflow	Framing and packing coils	1 (2)^b^	No	No	None	9/4/2023 (1.5 m/o)
13	Outflow, Sac, Inflow within 1 cm of sac	Framing and packing coils	1	No	No	None	9/18/2023 (13 days)

^a^Hemoptysis secondary to lung mass without evidence of PSA reperfusion

^b^Embolization of two separate lesions without recurrence

## Discussion

This study demonstrates the safety and efficacy of treating pulmonary artery pseudoaneurysms of various etiologies by embolizing the pseudoaneurysm sac with permanent embolic agents. All but two patients and cases were treated by embolizing the inflow pulmonary artery and pseudoaneurysm sac, and in most cases embolizing the pulmonary artery outflow as well. Previous studies evaluating mycotic aneurysms were treated with both pulmonary artery embolization and systemic-bronchial artery embolization. The rationale behind this treatment is the known phenomena of systemic-pulmonary fistulas in the setting of chronic lung inflammation [1, 6, 7]. However, in this study the bronchial arteries were neither interrogated nor embolized, including in the seven patients with mycotic aneurysm, chronic inflammatory lung disease (COPD with chronic bronchitis and bronchiectasis in both cases) and lung neoplasm for which systemic-pulmonary artery fistulas can arise. The rationale for treatment of the pseudoaneurysm sac, ideally with both inflow and outflow embolization is that any backflow from systemic arteries should be sufficiently prevented from filling the aneurysmal sac. Although the bronchial arteries do not routinely need to be interrogated if the aneurysm is identified on pulmonary angiography, careful attention to possible arteriovenous shunting must be made as this would preclude use of particle embolic agents. In this study no shunting was identified and PVA was safely used in three cases.

Pulmonary angiography generally requires pre-procedural imaging or a high suspicion for pulmonary arterial injury. In this study, eleven patients had pre-procedural imaging (11/13), five of which showed clear pseudoaneurysm on CTA and four demonstrating nonspecific contrast irregularity likely arising from the pulmonary arteries on routine CT. In two cases a large lung tumor with close involvement of the pulmonary artery was suggestive of the need to interrogate the pulmonary arteries rather than systemic arteries. In one case, a patient developed a pseudoaneurysm with active extravasation causing hypotension during a cardiac right heart catheterization. This was emergently treated by the interventional radiology team in the cardiac catheterization lab, obviating the need for pre-procedural imaging. Similarly, a second patient who was immediately post cardiac catheterization developed massive hemoptysis. This patient was immediately brought to the interventional suite with coil embolization of the pulmonary artery aneurysm inflow, sac, and outflow. However, shortly before closure the patient developed ventricular tachycardia and went into cardiac arrest for which resuscitation was unsuccessful.

Embolization of PAPs primarily with an inflow, outflow, and sac technique proved to be effective. There was only one case of minor recurrent hemoptysis secondary to reperfusion through a small, previously unseen, collateral pulmonary artery. This occurred one week after initial treatment and was retreated with PVA particles injected into the collateral pulmonary artery. There were no major complications and only two minor self-resolving episodes of pleuritis. There were two extenuating cases where the pseudoaneurysm sac was not embolized. One case already mentioned, an intraprocedural aneurysm with active extravasation occurred resulting in hypotension, necessitating rapid cessation of bleeding and a proximal embolization of the feeding artery was performed. The second case involved a large irregular PAP arising from the left main pulmonary artery, secondary to a large lung tumor, for which embolization of the sac was not technically feasible. In this case, PVA was injected distally to prevent back filling of the PAP, and a large Amplatzer plug was placed across the PAP.

Pulmonary artery pseudoaneurysms are both emergent and ominous findings. Of the ten patients who were treated at least one year prior to the date of this study, half of the PAPs (5/10) were noted to be deceased at the time of this study. The average survival period in these five patients was 7-months with a range of 0–14 months. More specifically, of the ten patients treated at least one year prior to the time of the study who presented with massive hemorrhage or hemoptysis, 5/6 were found to be deceased at the time of the study. All deaths were due to underlying disease, i.e., cardiac disease and lung neoplasms.

There are limitations of this study, the most significant being the small patient population in this study. Another limitation is the differentiation between true aneurysms and pseudoaneurysm. True peripheral pulmonary artery aneurysms and pseudoaneurysms typically cannot be differentiated based on imaging and are therefore assumed based on history. Little literature exists describing true aneurysms, with some managing the two entities as one^4^. However, an argument can be made for a watch and wait strategy when the lesions are small. There were two patients with asymptomatic lesions without a clear history suggestive of pseudoaneurysm or true aneurysm (i.e., congenital heart disease or connective tissue disease). Although, both patients had remote histories (late 90 s, childhood) of pneumonia, it is possible that these lesions may have represented true aneurysms and could have been followed without intervention. Treatment of true aneurysms are typically performed by embolizing the sac and inclusion of these two lesions may have therefore incorrectly increased the number of successful pseudoaneurysm embolizations utilizing this technique. However, given that the aneurysm type cannot be reliably differentiated by imaging and history alone, and that pseudoaneurysms are much more common than true aneurysms in the peripheral pulmonary arteries, these incidental lesions were treated in the same manner as known pseudoaneurysms.

## Conclusion

Pulmonary artery pseudoaneurysms are life threatening in the short term and an ominous finding with poor long-term prognosis in patients presenting with hemoptysis. Embolization of the pseudoaneurysm by occluding the inflow, outflow, and sac, is safe and effective in treating these lesions. Using this technique interrogation of the bronchial arteries may not be needed when the pulmonary artery origin can be clearly defined on pre-procedural imaging and/or is highly suggested by the clinical history.

## Data Availability

Data sharing is not applicable to this article as no data sets were generated or analyzed in the current study.
